# Dispersion Turning Attenuation Microfiber for Flowrate Sensing

**DOI:** 10.3390/s23167279

**Published:** 2023-08-20

**Authors:** Yaqi Tang, Chao Wang, Xuefeng Wang, Meng Jiang, Junda Lao, Dongning Wang

**Affiliations:** 1 Julong College, Shenzhen Technology University, Shenzhen 518118, China; tangyaqi@sztu.edu.cn (Y.T.);; 2 Institute of Beijing Aerospace Control Devices, Beijing 100094, China

**Keywords:** dispersion turning point, high attenuation fiber, anemometer, optical fiber sensor

## Abstract

We demonstrated a new optical fiber modal interferometer (MI) for airflow sensing; the novelty of the proposed structure is that an MI is fabricated based on a piece of HAF, which makes the sensitive MI itself also a hotwire. The interferometer is made by applying arc-discharge tapering and then flame tapering on a 10 mm length high attenuation fiber (HAF, 2 dB/cm) with both ends spliced to a normal single mode fiber. When the diameter of the fiber in the processing region is reduced to about 2 μm, the near-infrared dispersion turning point (DTP) can be observed in the interferometer’s transmission spectrum. Due to the absorption of the HAF, the interferometer will have a large temperature increase under the action of a pump laser. At the same time, the spectrum of the interferometer with a DTP is very sensitive to the change in ambient temperature. Since airflow will significantly affect the temperature around the fiber, this thermosensitive interferometer with an integrated heat source is suitable for airflow sensing. Such an airflow sensor sample with a 31.2 mm length was made and pumped by a 980 nm laser with power up to 200 mW. In the comparative experiment with an electrical anemometer, this sensor exhibits a very high air-flow sensitivity of −2.69 nm/(m/s) at a flowrate of about 1.0 m/s. The sensitivity can be further improved by enlarging the waist length, increasing the pump power, etc. The optical anemometer with an extremely high sensitivity and a compact size has the potential to measure a low flowrate in constrained microfluidic channels.

## 1. Introduction

An optical microfiber has many sensing applications, such as measuring the refractive index (RI) [1] and humidity [2], as the evanescent field on the surface of a microfiber easily interacts with the external environment [3]. The optical microfiber modal interferometer operating near the dispersion turning point (DTP) has been intensively investigated due to its ultra-high RI sensitivity [4,5]. The DTP is the point where the group effective RI difference of guide modes in coupling equals zero, and the RI sensitivity of the sensor can reach 10^4^ nm/RIU. For example, a tapered specially designed single stress-applying fiber is reported to have a maximum sensitivity of 30,563 nm/RIU [6]. Another highly sensitive gas refractometer shows a high temperature sensitivity of 2.134 nm/°C [7]. In addition, an optical microfiber coupler operating near the DTP successively senses gas and liquid RIs with an RI sensitivity up to 10^4^ nm/RIU [8,9]. Due to their excellent RI sensitivity, these sensors yield unusually brilliant results in other sensing fields as well [10].

In recent years, various types of optical fiber anemometers have been demonstrated, including hotwires [11,12,13], differential pressure [14], vibrations [15], and laser Doppler [16]. Among them, the optical fiber hotwire anemometer (HWA) is widely used [13,17], which can be either heated up via an electrical current [18] or pumping laser [19]. For example, an optical fiber anemometer based on a fiber Bragg grating (FBG) inscribed in a metal-filled micro-structured optical fiber showed a sensitivity of 0.091 nm/(m/s) with ultra-low pump power [20]. However, some of the optical fiber HWAs convert light energy into heat energy on the sensor surface through membranes such as silver [19] or graphene [21], etc., and the performance of such sensors is affected by membrane peeling and contamination. Because HAFs have strong absorption when the wavelength of the incident light is around 980 nm [22,23], there are also some types of optical fiber anemometers that use HAFs as the heat source. For example, the early implementations of airflow sensing was to write an FBG in a light absorption fiber, achieving a sensitivity of 0.083 nm/(m/s) within the wind speed range of 2–8 m/s. Subsequently, the sensitivity of the sensor to pump power was reduced by modulating the output power of the pump laser [24,25]. After that, a hotwire anemometer inscribing FBGs in HAFs and in standard telecom fibers was reported. The anemometer had a higher sensitivity of 0.445 nm/(m/s) at 0.5 m/s and showed a flowrate response at high temperatures [26]. In addition, there have been some reports of the use of HAFs to sense fluid flow, which indicates the good thermo-optical conversion performance of HAFs [27]. Both grating and interferometer-based self-heated fiber hotwire anemometers are promising approaches with high integration, fast response, and high resolution. In addition, these anemometers show higher sensitivity when the flowrate is smaller, and the sensitivity becomes lower as the flowrate increases [20,24,26].

Hotwire optical fiber anemometers generally measure flowrate by measuring the RI difference in the surrounding medium, and the optical microfiber-based modal interferometers operating near the DTP show their excellent RI sensitivity [4,5,6]. Due to the airflow taking away the surface temperature of the sensors, the surrounding RI changes and eventually leads to a wavelength shift. The ultra-high RI and temperature sensitivities of the dispersion turning attenuation microfiber could have a higher sensitivity in flowrate sensing. Here, a highly sensitive optical fiber anemometer based on an attenuation microfiber operating near the DTP is proposed. The attenuation microfiber can serve as the sensing element for monitoring the variation in the surrounding flowrate and, simultaneously, as a heat-generating element that converts the pump lights into heat. Such a sensor is ultra-highly sensitive to a flowrate of less than 1 m/s. More importantly, due to the spectral symmetry of the sensor, the sensitivity can be further enhanced to −2.69 nm/(m/s) at a flowrate of 1 m/s by tracing the separation between the twin dips on both sides of the DTP.

## 2. Sensing Principle of Dispersion Turning Attenuation Microfiber

**Figure 1:**
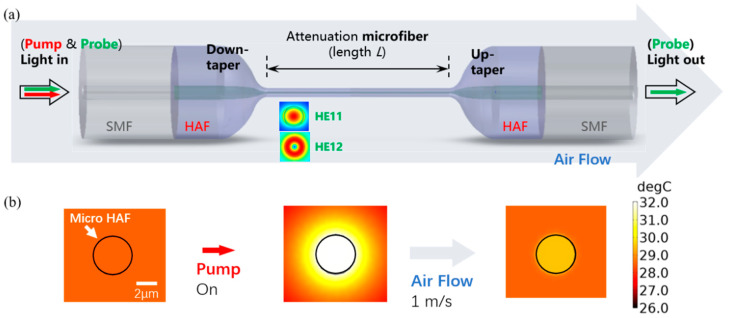
(**a**) Schematic diagram of the dispersion turning attenuation microfiber, (**b**) simulation results of dispersion turning attenuation microfiber for flowrate measurement.

Figure 1a shows the dispersion turning attenuation microfiber. It contains two abrupt transition regions and a central waist region, which has a significant fraction of evanescent fields. The fundamental mode (HE_11_) and the first high-order mode (HE_12_) are excited when the light transmits through the first abrupt transition regions. Due to the microfiber waist being about microns, the evanescent field of the excited modes leaks out of the microfiber and interacts with the ambient environment. When the ambient environment around a dispersion turning attenuation microfiber changes, the intensity of the output light that was originally in a steady state changes and stabilizes in another new state, and the changed output light can be expressed as follows [7]:

(1) $$I=I_{1}+I_{2}+2\sqrt{I_{1}I_{2}}\cos\left(\varphi_{N}\right)$$

where $I_{1}$ and $I_{2}$ are the intensities of HE_11_ and HE_12_, and $\varphi_{N}$ is the phase difference between the two modes. The periodic dips on the modal interference spectrum are associated with satisfaction of the following condition [28]:

(2) $$\varphi_{N}=\frac{2\pi }{\lambda_{N}}•L•\left(n_{eff}^{HE_{11}}-n_{eff}^{HE_{12}}\right)=\left(2N-1\right)•\pi$$

where $\lambda_{N}$ presents the wavelength of $N_{th}$ dip on the interference spectrum and L is the waist length of the attenuation microfiber. The performance of the anemometer is determined via the temperature changes in the sensor depending on the heat loss induced by wind flow. Therefore, the wavelength shift in the output light is the temperature change around the attenuation microfiber, and the temperature sensitivity of $S_{T}$ can be deduced as follows [8]:

(3) $$S_{T}=\frac{\lambda_{N}}{n_{g}^{HE_{11}}-n_{g}^{HE_{12}}}•\left(\frac{\partial \left(n_{eff}^{HE_{11}}-n_{eff}^{HE_{12}}\right)}{\partial T}+\alpha_{HAF}•\Delta n_{eff}\right)$$

where $n_{g}^{HE_{11}}$ and $n_{g}^{HE_{12}}$ are the group effective RIs of the HE_11_ and HE_12_ modes, respectively; $\alpha_{HAF}$ is the thermal expansion coefficient of the HAF; and $\partial \left(n_{eff}^{HE_{11}}-n_{eff}^{HE_{12}}\right)/\partial T$ is determined using the thermal-optic coefficient. The difference in group effective RIs between the HE_11_ and HE_12_ modes changes from negative to positive as the operating wavelength increases [7]. The sensitivity becomes highest when the difference of group effective RIs between the HE_11_ and HE_12_ modes is equal to zero, and the sensitivity reduced when the difference increases. So, the key factor in the emergence of the DTP is the fabrication of a micro optical fiber structure that can only pass through the HE_11_ and HE_12_ modes. Before conducting a dispersion turning attenuation microfiber flowrate measurement, the feasibility of HAFs is taken into consideration, as follows: firstly, the core and cladding diameter of the HAF are almost the same as that of SMF, which can reduce higher-order mode generation when both of them are fused together; secondly, the main effect of cobalt doped in the optical fiber core features a prominent reduction in power of the light transmission; and finally, the diameter of the fabricated dispersion turning attenuation microfiber is around a few microns, and the amount of cobalt in the thinner core is small, which hardly affects the output light.

When the sensor is heated by the pumping laser and thermal equilibrium is maintained, the airflow passing by the sensor will take away the heat and keep the sensor in a new thermal equilibrium. The process of breaking and maintaining the thermal equilibrium leads to a wavelength shift in the output signal, resulting in high performance of the dispersion turning attenuation microfiber [20]. Figure 1b shows the simulation results of the attenuation microfiber for a flowrate measurement with an initial ambient temperature of 20 °C, in which the position of the profile is 5 mm away from the down-tapered region, and the wind blows longitudinally along the optical fiber. It can be seen from Figure 1b that, when the pump power is transferred to the HAF and then keep at thermal equilibrium, the fiber and its surface have a temperature increase of about 12 °C compared with the surface temperature of the dispersion turning attenuation microfiber at the pump when power is off. Then, when a flowrate of 1 m/s is given along the direction of optical transmission, it can be found that the temperature of the optical fiber surface drops rapidly.

## 3. Dispersion Turning Attenuation Microfiber Fabrication

**Figure 2:**
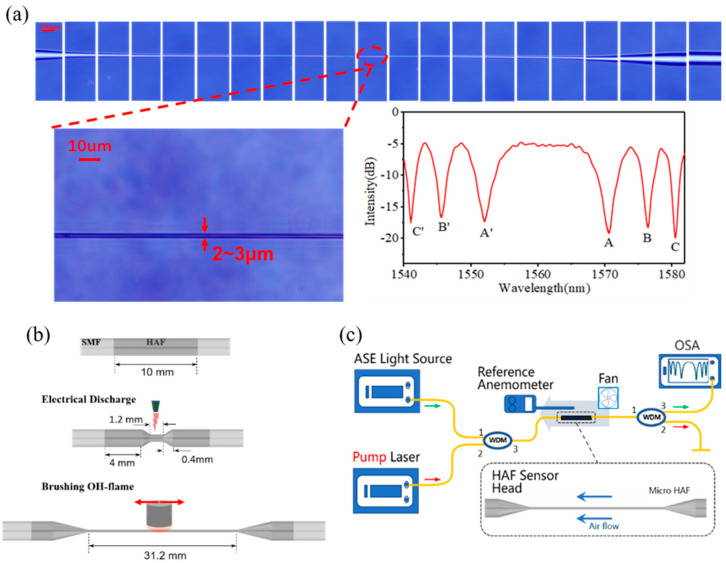
(**a**) Micrographs of fabricated attenuation microfiber and its transmission spectra, (**b**) fabrication process of attenuation microfiber, and (**c**) diagram of the sensing system.

The attenuation microfiber is fabricated by performing two tapering processes successively including electrode tapering and flame tapering. Electrode tapering aims to increase the steepness of the tapered area to make it easier to produce higher-order modes, while flame tapering aims to pull the surface of the sensor smoother and to make it easier to find the operating area near the DTP. The HAF used in the experiment was purchased from CorActive (HAF-CO1000-0180), with a wideband absorption coefficient of 2 dB/cm, and the SMF was from Corning Inc. In our study, the 10 mm length HAF was fusion-spliced to SMFs; then, the HAF was pre-tapered firstly by using LDS 2.5 (3SAE Technologies Inc., Franklin, MA, USA) with the following setting parameters: waist diameter, 75 μm; waist length, 1200 μm; and tapered length, 400 μm. Then, the pre-tapered HAF was further tapered by using an optical fiber fusion taper machine. The manufacturing process of the attenuation microfiber is shown in Figure 2b. The top of Figure 2a shows the prepared graph of attenuation microfiber; it can be seen that there is an abrupt reduction in diameter on both sides of the HAF, which helps to generate a higher-order mode of the waist microfiber region. The waist with a diameter of 2~3 μm, which allows only the transmission of the HE_11_ and HE_12_ modes, is shown in the lower left of Figure 2a. As shown in the lower right of Figure 2a, the transmission spectrum is obtained when the total lengths of the attenuation microfiber and DTP are ~31.20 mm and ~1560 nm, respectively, and it can be found that the HE_11_ and HE_12_ modes were both generated.

## 4. Experiments and Analysis

The experimental setup of the sensing system is shown in Figure 2c: a broadband source (BBS) and a pump laser at 980 nm are coupled into the system via a wavelength division multiplexer (WDM) by connecting port 1 and port 2, respectively; then, the WDM combines and transfers the light produced from the pump laser and BBS to the incident port of the attenuation microfiber; and in order to prevent damage to the optical spectrum analyzer (OSA, AQ6374), the high-power heating laser is split by connecting another WDM to the transmission port of the attenuation microfiber. The output spectrum was monitored using an OSA with a wavelength resolution of 0.05 nm.

**Figure 3:**
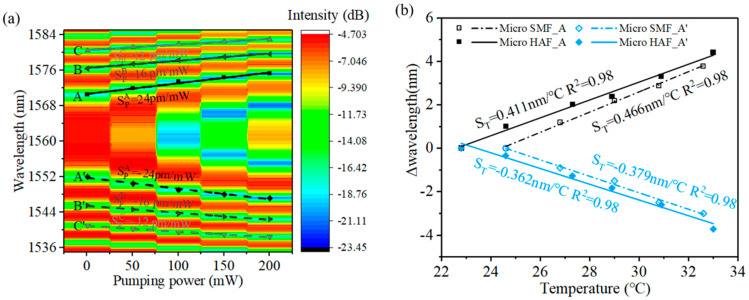
(**a**) Wavelength shift in different dips with different pump power, and Si means the sensitivity of the interference dips. (**b**) Wavelength shifts in interference dips A and A’ of attenuation microfiber and micro SMF with different temperatures.

The relationship between the pump power and transmission spectrum of the attenuation microfiber is shown in Figure 3a. It is found that the interference dips drifted away from the DTP when the pump power rose from 50 mW to 200 mW with an interval of 50 mW. According to Reference [7] and Equation (3), the sensor dips would shift with ambient parameter changes, for example, changed in the temperature and RI. When the pump power increases, the HAF will generate more heat, resulting in a redistribution of local temperature and RI around the HAF. So, the interference dips drift away accordingly. The results show equal sensitivity of the dips that are symmetrically located on both sides of the DTP. And, it can be seen that, when the interference dip is closer to DTP, the corresponding sensitivity is higher. The highest sensitivity responses to the pump power of dips A and A’ are 24 pm/mW and −24 pm/mW, respectively. Because the pump laser firstly passes through a 4 mm HAF before being directed to the attenuation microfiber, the actual pump power corresponding to the dip wavelength is smaller than the measuring pump power. Further analyses could take into consideration that the length of the HAF used to fabricate the attenuation microfiber is only 2 mm, which means that the pump power used to heat the attenuation microfiber inducing the wavelength shift is only ~8% (which means the actual sensitivity of the pump power is 0.338 nm/mW) and photothermal efficiency could be improved by an order magnitude.

Then, the temperature response of the attenuation microfiber was measured and is shown in Figure 3b. It can be seen that the temperature sensitivities of an attenuation microfiber and a common microfiber on dips A and A’ have similar values around 0.4 nm/°C. So, when the pump power is 200 mW, the temperature rise in the microfiber rises can be estimated to about 13 °C from the wavelength shift. The pumped temperature rise makes the attenuation microfiber a good candidate for measuring flowrate [22].

**Figure 4:**
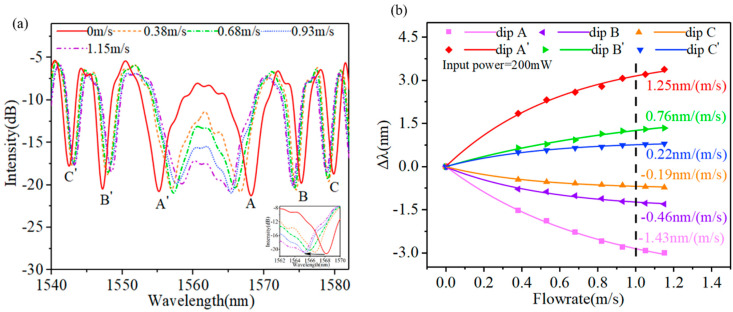
(**a**) Variation in the transmission spectrum of an attenuation microfiber with different flowrates. (**b**) Wavelength shifts in interference dips with different flowrates.

The dispersion turning attenuation microfiber is then used for flowrate measurement, as shown in Figure 2c. The attenuation microfiber is susceptible to vibration interference caused by transverse wind, which will increase the noise, which is not conducive to measuring flowrate. In the experiment, the wind blows longitudinally along the optical fiber. Figure 4a reveals the wavelength shift in the attenuation microfiber with different flowrates. In the experiment, the pump power is set at 200 mW. The higher the flowrate, the more heat will be taken away around the dispersion turning attenuation microfiber. Thus, it is found that the interference dips/peaks located on both sides of the DTP shift toward each other as the flowrate increases. And, the interference dips/peaks drifts more when they are closer to the DTP.

To more intuitively display the proposed anemometer sensor’s sensitivity, the wavelength difference in dips A, A’, B, B’ C, and C’ between different flowrates and no wind was obtained and is shown in Figure 4b. The dips on both sides of the DTP present the negative and positive sensitivities, respectively. The fitted curve satisfies the formula y=A×e^(−x/C)+y0, where y and x present the ∆λ and flowrate, respectively; A and C are the flowrate coefficients of the interference dips, related to flowrate sensitivity; and the flowrate sensitivity can be deduced from S=−A/C×e^(−x/C). Figure 4b shows the dip sensitivities at the flowrate of 1 m/s, dips A and A’ in these dips are the highest and show −1.43 nm/(m/s) and 1.25 nm/(m/s), respectively, which indicates that the temperature went down by about 6.8 °C. The sensitivities of dips B, C, B’, and C’ are −0.46 nm/(m/s), −0.19 nm/(m/s), 0.22 nm/(m/s), and 0.76 nm/(m/s), respectively. More interestingly, the twin dips that are symmetrically located with respect to the DTP shift toward each other as the flowrate increases. This unique characteristic of the twin dips provide a new method for measuring flowrate by tracing the separation between the twin dips, which could further increase the flowrate sensitivity [7]. The twin dips AA’ provide the highest flowrate sensitivity of −2.69 nm/(m/s), which is the sum of the sensitivities of dips A and A’.

**Figure 5:**
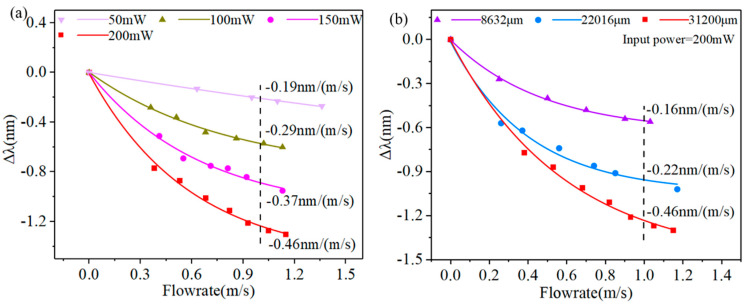
(**a**) Variation in the transmission spectrum of an attenuation microfiber with different flowrates. (**b**) Wavelength shifts in interference dips with different total lengths of an attenuation microfiber.

Figure 5a reveals the dependence of the dip B wavelength on the flowrate with different pump powers. It is found that when the pump power gradually increases, the sensitivity of dip B increases too. Because the increase in pump power allows the attenuation microfiber to absorb more light energy, which generates more heat, the temperature increases for the microfiber surface. When the same airflow passes through the attenuation microfiber, more heat is lost on its surface, which results in a fast temperature drop. So, the sensitivity of the attenuation microfiber is higher. Figure 5b shows the sensitivities of the optical fiber anemometer of dip B under 200 mW pump power with the total length of the attenuation microfiber at 8632 μm, 22,016 μm, and 31,200 μm. It can be found that, when the flowrate is 1m/s, the sensitivities can reach −0.16 nm/(m/s), −0.22 nm/(m/s), −0.46 nm/(m/s), respectively, which shows that the flowrate response of the optical fiber anemometer is enhanced with the increase in attenuation microfiber waist length. The reason for this phenomenon may be due to the fact that the attenuation microfiber that produces the DTP becomes longer, leading to an increase in the heat dissipation surface area; hence, the dissipation capability of the sensor is stronger and has a higher sensitivity.

The pump-induced wavelength-change shown in Figure 3a can be used to adjust the dip wavelength and to overcome ambient temperature crosstalk: Firstly, a temperature T_0_ is chosen as the initial temperature, and λ_0_ is the corresponding dip wavelength of the interference. Then, at a different environmental temperature T, the dip wavelength λ(T) is moved to λ_0_ by changing the pump power. Next, the flowrate coefficients A and C(T) of the sample are measured and recorded at different T but from the same λ_0_. When performing a practical flowrate test, the environment temperature T_x_ can be derived from the pre-tested λ(T) relation or by using an extra FBG, so that the corresponding sensitivity coefficients A and C(T_x_) can be obtained from the record.

Table 1 summarizes the key parameters of some reported optical fiber hotwire anemometers. From Table 1, we can conclude that our sensor exhibits the highest flow sensitivity in the comparison, which is about an order of magnitude improvement. The higher sensitivity comes from the increase in operation temperature for the attenuation microfiber and the extremely high temperature sensitivity of the dispersion turning attenuation microfiber. It is worth noting that the novelty of this paper is the proposed high sensitivity of an all-fiber interferometer based on HAFs. To demonstrate the benefits of using HAFs, we build an airflow sensing system as an example application. In this paper, the samples for flowrate sensing exhibit an extremely high sensitivity but small dynamic range. This can be improved by increasing the pump power, using HAFs with high attenuation coefficients, and optimizing the HAF length. Potential applications for this sensor may lie in flow analysis in constrained spaces such as microfluidic systems. The all-optical sensing principle makes this sensor available in extreme environments such as strong electromagnetic interference, high temperature, and corrosion, in which existing electrical anemometers find difficulty. In addition, this structure can be used for liquid flowrate sensing and building light control devices.

## 5. Conclusions

In conclusion, we experimentally demonstrated that an attenuation microfiber operating near the DTP can be used to measure flowrate. This anemometer provides an excellent ability to measure flowrate response. As a result, the sensitivity can reach −2.69 nm/m/s at the flowrate of 1 m/s, and the sensitivities are higher at lower flowrates. The capability to enhance the flowrate sensitivity of an attenuation microfiber has been investigated by increasing pump power, using HAFs with high attenuation coefficients, and optimizing HAF lengths. Since this anemometer shows higher sensitivity at a lower flowrate, it has potential in micro airflow measurement applications.

## Figures and Tables

**Table 1:** Performance of some reported optical fiber anemometers.

Type	Heat Source	Sensitivity (nm/(m/s))	Range (m/s)	Pump Power (mW)	Reference
TFBG	Single-walled carbon nanotube	−0.3667 at 1 m/s	0–2.1	97.76	[11]
TFBG	Carbon nanotube	0.898	0.05–0.65	10	[12]
45°-TFBG + FBG	Single-walled carbon nanotube	0.14 at 1 m/s	0–1	20	[28]
FBG + no core fiber	Silver	0.5915 at 0.68 m/s	0–13.7	340	[19]
Elliptical core micro-FBG	Graphene	0.42	0–1	400	[21]
FPI	Ultraviolet-cured adhesive	−0.91 at 1 m/s	0–7	100	[29]
FPI	Sn	−0.83 at 1 m/s	0–10	45	[30]
FBG	HAF	0.083	2–8	299.3	[24]
Regenerated FBG	HAF	0.445 at 0.5 m/s	0–0.66	700	[26]
Our work	Dispersion turning attenuation microfiber	−2.69 at 1 m/s	0–1.1	200	

## Data Availability

Not applicable.
